# Assessing the impact of a new health sector pay system upon NHS staff in England

**DOI:** 10.1186/1478-4491-6-12

**Published:** 2008-06-30

**Authors:** James Buchan, David Evans

**Affiliations:** 1 Queen Margaret University, Edinburgh, Scotland, EH21 6UU, UK; 2 Kings Fund, Cavendish Square, London, W1G 0AN, UK; 3 Capita Health Services Partners, Harrogate, North Yorkshire, HG1 5PR, UK

## Abstract

**Background:**

Pay and pay systems are a critical element in any health sector human resource strategy. Changing a pay system can be one strategy to achieve or sustain organizational change. This paper reports on the design and implementation of a completely new pay system in the National Health Service (NHS) in England. 'Agenda for Change' constituted the largest-ever attempt to introduce a new pay system in the UK public services, covering more than one million staff. Its objectives were to improve the delivery of patient care as well as enhance staff recruitment, retention and motivation, and to facilitate new ways of working.

**Methods:**

This study was the first independent assessment of the impact of Agenda for Change at a local and national level. The methods used in the research were a literature review; review of 'grey' unpublished documentation provided by key stakeholders in the process; analysis of available data; interviews with key national informants (representing government, employers and trade unions), and case studies conducted with senior human resource managers in ten NHS hospitals in England

**Results:**

Most of the NHS trust managers interviewed were in favour of Agenda for Change, believing it would assist in delivering improvements in patient care and staff experience. The main benefits highlighted were: 'fairness', moving different staff groups on to harmonized conditions; equal pay claim 'protection'; and scope to introduce new roles and working practices.

**Conclusion:**

Agenda for Change took several years to design, and has only recently been implemented. Its very scale and central importance to NHS costs and delivery of care argues for a full assessment at an early stage so that lessons can be learned and any necessary changes made. This paper highlights weaknesses in evaluation and limitations in progress. The absence of systematically derived and applied impact indicators makes it difficult to assess impact and impact variations. Similarly, the lack of any full and systematic evaluation constrained the overall potential for Agenda for Change to deliver improvements to the NHS.

## Background

Pay and pay systems are a critical element in any health sector human resource strategy. Pay rates are a factor in determining how the organization connects with external labour markets, through staff recruitment and retention, and the type of pay system selected by an organization can be a major factor in creating organizational culture and supporting specific types of staff behaviour and performance. Changing a pay system can be one strategy to achieve or sustain organizational change. This paper, commissioned by the Kings Fund, reports on the design and implementation of a completely new pay system in the National Health Service (NHS) in England.

In this paper progress in implementing "Agenda for Change", the new pay system for NHS staff, is examined. Agenda for Change constitutes the largest-ever attempt to introduce a new pay system in the UK public services, covering more than one million staff. Its objectives were to improve the delivery of patient care and support new ways of working, as well as to enhance staff recruitment, retention and motivation.

The primary objectives of the research were firstly to examine the impact of the new pay system at operational level, through the case studies in the 10 trusts, where data and information was sought on changes in costs, systems and staff behaviour (e.g. turnover, absence rates); secondly to assess the national situation through analysis of national level data and information on costs and impact; and thirdly to assess the relevance and effectiveness of any systematic evaluation of impact that was being conducted. As such the study was intended to provide both local level detail of the process of implementation, and national level key findings [1]. The main audience for the research was national level policy makers and local level management.

### The case for change in NHS pay

By the mid-1990s, the NHS pay system, developed nearly 50 years earlier with the creation of the NHS in 1948, was increasingly being seen as outdated and not fit for purpose. It was based on national bargaining units ("Whitley councils"), each involving multiple staff associations/trade unions representing different staff groups. The Whitley system was regarded by many as complex and inflexible, constraining the development of new roles and unresponsive to the high levels of contribution being made by experienced clinical staff. It was also open to challenge on the basis of equal pay for work of equal value. Pressure to overhaul the pay system was growing as the century ended.

With the election of a Labour government in May 1997, the prospect of a new NHS pay system was raised. The new government's White Paper on Health, published at the end of 1997, announced the intention to 'modernise' the NHS: 'In a national health service, the current mix of national and local contracts is divisive and costly. The Government's objective for the longer term was therefore to see staff receive national pay, if this could be matched by meaningful local flexibility, since the existing national terms of service for a multitude of staff groups were regarded as inequitable and inflexible [2].

In February 1999, the government published its proposals for a new pay framework for NHS staff, "Agenda for Change – Modernising the NHS Pay System" [3]. The proposals included simplified national pay 'spines' covering different staff groups, a national job evaluation scheme and a competency-based career framework (later named the Knowledge and Skills Framework (KSF)). The proposals emphasized that the new system was designed to:

• enable staff to give their best for patients, working in new ways and breaking down traditional barriers;

• pay fairly and equitably for work done, with career progression based on responsibility, competence and satisfactory performance;

• and simplify and "modernise conditions of service, with national core conditions and considerable local flexibility.

Agenda for Change was just one element in the overall approach to modernising the NHS and introducing a new approach to workforce policy and planning. An NHS human resources strategy for workforce expansion and new ways of working was adopted [4], and a blueprint for establishing a new approach to workforce planning and development was agreed [5]. The new pay systems for NHS staff were regarded as critical, integral elements in this process of change.

The initial plan was to reach agreement with NHS staff associations and trade unions on the new system by September 1999. This target date proved to be hopelessly optimistic. In December 2002 an 'understanding' was finally reached between the national negotiators from management and unions and a framework document was published. Negotiations continued and the proposed agreement, including a three-year pay deal, was published on 28 January 2003. Implementation began with a piloting process in 12 'early implementer' sites, followed by the national roll-out of Agenda for Change from 1 December 2004. By the end of 2006, more than 99% of staff in England was on Agenda for Change pay arrangements (see Table 1).

**Table 1:** The implementation timetable for Agenda for Change.

May 1997	Labour government elected
September 1997	Exploratory talks on a new NHS pay system begin
December 1997	White Paper on modernising the NHS is published
February 1999	*Agenda for Change- Modernising the NHS Pay System *is published
October 1999	First joint statement of progress
November 2000	Second joint statement of progress
November 2001	Third joint statement of progress
December 2002	Framework agreement agreed and published
January 2003	Proposed agreement and three-year pay deal announced
June 2003	'Early implementer' sites begin to implement Agenda for Change in England
December 2004	National roll-out of Agenda for Change starts in England
September 2005	Original deadline for assimilating staff on to new pay and conditions
October 2006	Original deadline for implementation of Knowledge and Skills Framework
February 2007 – April 2007	Consultation on draft proposals for unsocial hours payments
2007	Full implementation (other than ongoing consultation on new unsocial hours payments)

Source: Buchan and Evans 2007 [1]

### What is Agenda for Change?

Agenda for Change has been the largest and most ambitious attempt ever to reform the NHS pay system. The new pay system applies to more than 1 million NHS and it covers all staff groups apart from doctors and dentists, who have separate new pay contracts, and very senior managers, who are mainly employed on individual contracts of employment. Table 2 gives the key features of the new system.

**Table 2:** Key elements of Agenda for Change: pay bands (April 2006).

Pay band	Job weight	Pay range at 1 April 2006
1	0–160	£11 782 to £12 853
2	161–215	£12 177 to £15 107
3	216–270	£14 037 to £16 799
4	271–325	£16 405 to £19 730
5	326–395	£19 166 to £24 803
6	396–465	£22 886 to £31 004
7	466–539	£27 622 to £36 416
8a	540–584	£35 232 to £42 278
8b	585–629	£41 038 to £50 733
8c	630–674	£49 381 to £60 880
8d	675–720	£59 189 to £73 281
9	720–765	£69 899 to £88 397

Each pay band consists of a number of pay points, and staff progress from point to point on an annual basis to the top point of their pay range or pay band, provided their performance is satisfactory and they can demonstrate the agreed knowledge and skills appropriate to that part of the pay range or band.

There are special arrangements for new entrants to band 5 [1].

Agenda for Change introduced two new pay spines: one for nurses and other health professionals; and one for other directly employed NHS staff. These two pay spines replaced the multiplicity of occupational pay grades, pay points and salary scales that had characterized the Whitley system.

To ensure that 'equal pay for work of equal value' was delivered, the pay system was underpinned by a job evaluation scheme, which was based on 16 factors. Each factor (e.g., 'analytical and judgement skills', 'emotional effort' and 'working conditions') had different identified levels, and a points score was derived for each job. The factors and the weighting and scoring system used in Agenda for Change were developed as a tailor-made system for NHS staff as it was agreed there was no pre-existing system capable of evaluating all of the jobs covered.

The new pay spines are divided into nine pay bands, and staff covered by Agenda for Change were assimilated on to one of these pay bands on the basis of job weight, as measured by the NHS job evaluation scheme.

Agenda for Change also harmonized terms and conditions of employment:

• Standard working hours for full-time staff of 37.5 hours a week, excluding meal breaks, although protection and assimilation arrangements mean that this will not be fully achieved until December 2011.

• Single harmonized rate of time-and-a-half for all staff in pay bands 1 to 7 eligible for overtime payments, and double time for overtime on general public holidays.

• Annual leave entitlement (excluding 8 public holidays) of 27 days on appointment, rising to 29 days after 5 years' service and to 33 days after 10 years

See [1] for details.

A critical element in Agenda of Change is the Knowledge and Skills Framework (KSF). This provides a framework for the review and development of each staff member and is the basis for determining individual employee pay and career progression within Agenda for Change. Each job has a KSF post outline that sets out the dimensions, levels and indicators required for the post-holder to undertake their job effectively. The KSF process is based on an annual developmental review between each staff member and their line manager, which should produce a personal development plan (PDP) (for details see [6]). The recent Health Committee Report on NHS Workforce Planning concluded that 'Effective use of the KSF has great potential to improve staff productivity. The KSF can improve access to relevant education and training, and support amended roles which will allow staff to develop the skills required to increase flexibility and efficiency" [7].

## Methods

This study was the first independent assessment of the impact of Agenda for Change at a local and national level. The methods used in the research were a literature review; review of 'grey' unpublished documentation provided by key stakeholders in the process; analysis of available data; interviews with key national informants (representing government, employers and trade unions), and case studies conducted with senior human resource managers in ten NHS hospitals in England [1].

The detailed case studies were conducted in NHS hospital "trusts" in England from late March 2007 to May 2007 (see Table 3 for details). Interviews were conducted with senior HR managers in each trust, using a standard interview schedule. The schedule covered six areas: a review of the implementation process so far in the trust; reported experience so far in meeting five key improvement themes related to Agenda for Change (benefits realization, financial management, strategic fit, redesign & modernisation, value for money); specific detail on trust level work on benefits realization; the local timeline to achieve full benefits; the main indicators being used locally to evaluate the impact of Agenda for Change; and future plans for implementation/utilization of Agenda for Change.

**Table 3:** Case study NHS hospital trusts, March-May 2007.

Case study 1	teaching hospital in the South East
Case study 2:	acute specialist trust in the North West
Case study 3	acute trust in the South East
Case study 4	acute trust in Yorkshire & Humberside
Case study 5	acute trust in the North West
Case study 6	acute hospital in the South East
Case study 7	acute teaching trust in London
Case study 8	teaching trust in London
Case study 9	acute trust in the South West
Case study 10	acute trust in the South West

Interviews were also conducted with key national informants who had been involved in the national negotiations and/or have a current policy responsibility for NHS pay. These interviewees came from both management and union/professional associations, and in most cases they were interviewed twice; once before the local case studies had been conducted, and again afterwards.

## Results

This section reports on the findings from the case study hospital trusts in terms of the reported experience so far in implementing Agenda for Change, and from feedback from key national informants.

### The rationale for Agenda for Change

Most of the NHS trust managers interviewed were in favour of Agenda for Change, believing that, in part at least, it would assist in delivering the improvements in patient care and staff experience that were its stated objectives. The main benefits of Agenda for Change highlighted by these managers were: 'fairness', moving different staff groups on to harmonized conditions; equal pay claim 'protection'; and scope to introduce new roles and working practices.

### Costs

Implementing a new pay system inevitably incurs costs – both one-off costs linked to the process of setting up new systems, and ongoing costs if staff are assimilated on to the new structure at a higher level. Given the need to account for and control these costs and to check actual costs against planned (and funded) pay changes, it is surprising that not all the case study trusts could provide a detailed assessment of local costs of implementation, and those that did provided different types of costing. Three trusts provided cost estimates of '3.6% ', 'about 2.5% to 3%', and 'between 4% and 6%' on the pay bill in the first full year of implementation. The absence of a detailed costing by all the trusts, and the absence of a consistent approach to costing, provides one example of the relative lack of detailed evaluation of impact of Agenda for Change. Many managers also reported that the additional funding provided had not been sufficient to cover the estimated cost of implementation.

At national level the absence of any full evaluation of implementation of Agenda for Change has limited an assessment of its costs and benefits. Of the total 43 billion UK sterling cash increase in NHS spending over the period 2002/3 to 2007/8 it has been estimated that 43% (18.9 billion UK sterling) has been absorbed in higher pay and prices – mainly pay increases under Agenda for Change and for medical staff. The implementation costs for the new pay system have been calculated as a cumulative additional cost of 2 200 million UK sterling in 2005/6 to 2008/9 [8].

### Implementation

Agenda for Change represents a new approach to pay determination for NHS staff. One critical factor that impacted during the latter stages of implementation was that the financial situation facing the NHS was much tighter than was the case when Agenda for Change was being developed. The long delays in negotiation and implementation meant that the new pay system was beginning to function just as the NHS in England has moved from a period of relative funding growth to one of fiscal constraint, and where there has been greater scrutiny on public sector pay awards [9-11].

The timing of implementation took longer than anticipated, and coverage was not complete at the time of the case study research. At the time of the case studies, from March to May 2007, none of the managers interviewed reported that their organization had yet achieved 100 per cent staff coverage of personal development plans (PDPs) or had all their relevant staff assimilated on to the KSF. They reported between 60% and 'nearly all' staff on PDPs; and from 'not yet all' staff, up to '95%' and 'virtually all' staff being on KSF job outlines. Managers in the case study hospitals highlighted the fact that full benefits realization is not achievable without a fully functioning KSF: 'We need to maintain focus to fully embed KSF and maintain the integrity of the system'; 'The key challenge now is getting KSF sorted'.

These findings are supported by the results of a national survey which quoted figures gathered by SHAs in December 2006 [12] suggesting that at that time only 67 per cent of staff have a full KSF job outline.

### Impact

Agenda for Change was intended to be a means to an end – to facilitate the development of new roles and new ways of working, and to improve staff recruitment and retention. This so-called 'benefits realization' was highlighted as the rationale for investing in the new pay system.

The Department of Health in England published a draft benefits realization framework in October 2004 to help NHS organizations deliver the benefits expected of Agenda for Change which made it clear that Agenda for Change would be 'a contributory factor to achieving the success criteria rather than the sole factor' [13]. The framework included detailed suggestions on approaches to measurement and data sources to be used. This was followed by a schedule and timeline for benefits realization [14] (see Table 4).

**Table 4:** NHS Employers benefits timeline for Agenda for Change.

**Implementation benefits**	**Intermediate benefits**	**Long-term benefits**
Fair pay	More teamwork	More patients treated
Better pay	Greater innovation in staff deployment	Higher-quality care
Partnership working	Better career development	
Equal opportunities and diversity	Better recruitment and retention	
Human resources systems	Better morale	
Simplified administration		

(NHS Employers, 2005)[14]

At the time of the research, Agenda for Change had been implemented for about a year in the case study trusts. At this relatively early stage in the process, most of the managers interviewed could identify positive changes that had already been achieved within their hospital as a result of Agenda for Change. They all said their hospital trusts were either in the implementation or intermediate phase of benefits realization, as outlined in the benefits timeline.

Four main areas of 'implementation benefits' were identified by managers as having already been achieved in most of the hospital trusts. These were: HR systems (e.g. improved job descriptions), better partnership working (e.g. more effective management-trade union relations), equal pay and simplified human resources/payroll administration.

The timeline for achieving benefits realization included two long-term benefits: 'more patients treated more quickly' and 'higher-quality care'. Managers in the case study trusts indicated that they believed it would take another two to five years to achieve these long-term benefits. Even then, several cautioned that the broader impact of financial deficits and tightening of NHS funding streams, combined with the knock-on effects of increased pay bill costs as Agenda for Change was implemented fully, meant that full benefits realization would be challenging and problematic.

This was echoed at national level by some of the interviewees, one union official commenting that: 'The jury is out on benefits realization. There are good examples of trusts using Agenda for Change to bring about improvements in care, but the mainstream NHS has so far failed to grasp the challenge... Without further central government pressure to deliver, opportunities will be lost.'

Some additional evidence of the impact of Agenda for Change can be found in staff surveys. The October 2006 NHS staff survey conducted by the Healthcare Commission included, for the first time, some questions directly concerning the implementation of Agenda for Change [15].

Almost 69 500 staff from 171 NHS hospital trusts took part in this survey. Nearly three quarters of staff in acute trusts reported receiving a new job outline or job description and some 35% agreed or strongly agreed that they were satisfied with the information they received from their trust about Agenda for Change; 29% disagreed or strongly disagreed (Table 5).

**Table 5:** Responses of staff in acute trusts in relation to survey questions about Agenda for Change.

Agenda for Change	Yes or agree/ strongly agree	No or disagree/ strongly disagree	Do not know or neither agree/disagree
Pay banding is fair	41%	35%	15%
Implemented successfully	25%	33%	36%
Has resulted in taking on increased responsibilities in job	21%	35%	32%

Source : The Healthcare Commission (2007) [15]

The annual survey of nurses conducted by the Royal College of Nursing reported similar findings: many nurses reported that they did not believe that the job evaluation process had been carried out well at local level [16]. Many, but not all of the nurses, believed they would be better treated under Agenda for Change. In all, 44 % of nurses reported that they thought they would be better off under Agenda for Change, while 37% believed their circumstances would not change and 12% thought they would be worse off.

## Discussion

The key finding of the case study research at local level was that, while interviewees could point to local examples of benefits realization, there has so far been only limited evaluation of the experience of implementation and of the impact of Agenda for Change. The results of the case study research highlighted variable local impact in the ten case study NHS hospital trusts, variation in local assessment of costs of implementation, and an absence of systematic national or regional level monitoring of impact. Results of national staff surveys highlighted a mixed picture of impact, and suggested that implementation had not been 'felt fair' by many staff. The results from 10 case studies hospitals cannot be extrapolated to the whole of an organization of several hundred hospitals. Despite the huge overall costs, there has been no systematic assessment of costs, benefits and impact. (The same criticism has been made about the implementation of new pay contracts for hospital consultants and for general practitioners) [17,18].

## Conclusion

The paper has set out an assessment of progress up to mid-2007 with the implementation of the new pay system within a national health system. Agenda for Change is the largest-ever attempt to develop a new ('modern') pay system in the public services in the United Kingdom. It affects the livelihood of more than 1 million workers, has a major impact on NHS finances, and by introducing links to the knowledge and skills of the workforce, it also affects patient care. The case study research reported in this paper report was the first independent assessment of the implementation and impact of the new pay system, and highlights weaknesses in evaluation and limitations in progress. The limited evidence made available in the case studies and from staff surveys shows some positive changes are occurring as a result of the new pay system, but that the impact is variable between local level NHS trusts. The absence of systematically derived and applied impact indicators makes it difficult to assess impact and variations in impact across the NHS.

While the pay system implemented in the NHS was designed for the characteristics of that health care organization, there are some more general lessons for any country or health system considering a significant change in their approach to pay determination. The time taken to negotiate, design and implement the new pay system (several years) reflects the complexities of the process, but also the need to reconcile the changing and sometimes conflicting demands of various national stakeholders- government departments, trade unions, employers, etc. While sufficient time must be built into such a process to accommodate these requirements, the longer the process, the greater the danger that the organizational context and priorities in which the pay system will function may have changed. In the case of the NHS this was most notable in relation to the changing funding situation across the time period. Another key point is that achieving implementation of a new pay system ('ticking the box') should not be regarded as the end of the process. It may take years before a new pay system delivers on some of its stated objectives; and if there is inadequate evaluation, this can hinder assessment of progress made in delivery of these longer term objectives.

It could be argued that it is "early days" for Agenda for Change – it took several years to design, and has only recently been implemented. But its very scale and central importance to NHS costs and delivery of care argues for a full assessment at an early stage so that lessons can be learned and any necessary changes made. Given the scale of the exercise, its costs and assumed benefits, the absence of any full and systematic evaluation constrains the overall potential for Agenda for Change to deliver improvements to the NHS.

## Competing interests

The authors declare that they have no competing interests.

## Authors' contributions

JB Directed the study, contributed to design, methods, fieldwork and report writing, DE contributed to design, methods, fieldwork and report writing.
